# Structural insights into DNA cleavage activation of CRISPR-Cas9 system

**DOI:** 10.1038/s41467-017-01496-2

**Published:** 2017-11-09

**Authors:** Cong Huai, Gan Li, Ruijie Yao, Yingyi Zhang, Mi Cao, Liangliang Kong, Chenqiang Jia, Hui Yuan, Hongyan Chen, Daru Lu, Qiang Huang

**Affiliations:** 1 State Key Laboratory of Genetic Engineering, School of Life Sciences, Fudan University, Shanghai, 200438 China; 2 National Center for Protein Science Shanghai, Institute of Biochemistry and Cell Biology, Shanghai Institutes for Biological Sciences, Chinese Academy of Sciences, Shanghai, 200031 China; 3 Shanghai Science Research Center, Chinese Academy of Sciences, Shanghai, 201204 China

## Abstract

CRISPR-Cas9 technology has been widely used for genome engineering. Its RNA-guided endonuclease Cas9 binds specifically to target DNA and then cleaves the two DNA strands with HNH and RuvC nuclease domains. However, structural information regarding the DNA cleavage-activating state of two nuclease domains remains sparse. Here, we report a 5.2 Å cryo-EM structure of Cas9 in complex with sgRNA and target DNA. This structure reveals a conformational state of Cas9 in which the HNH domain is closest to the DNA cleavage site. Compared with two known HNH states, our structure shows that the HNH active site moves toward the cleavage site by about 25 and 13 Å, respectively. In combination with EM-based molecular dynamics simulations, we show that residues of the nuclease domains in our structure could form cleavage-compatible conformations with the target DNA. Together, these results strongly suggest that our cryo-EM structure resembles a DNA cleavage-activating architecture of Cas9.

## Abstract (web summary)

CRISPR-Cas9 is widely used for genome engineering but structural data for the DNA cleavage step are still incomplete. Here, the authors present the cryo-EM structure of a ternary Cas9-sgRNA-target DNA complex, perform MD simulations and discuss implications for the Cas9 DNA cleavage mechanism.

## Introduction

The genome editing process by the CRISPR-Cas9 system^1, 2^ is mediated by a single-guide RNA (sgRNA) and consists of two steps: DNA binding and DNA cleavage. In brief, the sgRNA-guided Cas9 first binds to the target DNA by interacting with its protospacer adjacent motif (PAM); then, the two nuclease domains (HNH and RuvC) catalyze the splitting of the scissile bonds in two DNA strands, respectively^3, 4^. Several studies^5–7^ reported the ternary complex structures of Cas9, sgRNA, and target DNA, and elucidated how Cas9 binds to its target DNA. Also, it is known that the HNH domain cuts the 20-mer target sequence complementary to sgRNA, and the RuvC domain cleaves the non-target sequence^8, 9^.

Structural studies were essential for understanding the molecular mechanism of DNA binding to the bilobe-like Cas9–sgRNA binary complex^5–7, 10^. However, structural information about the DNA cleavage step are incomplete. For example, the HNH active site in the earlier crystal structures is at a position far from the cleavage site of the target strand (>32 Å; HNH-state 1 in Supplementary Fig. 1a, b)^6, 7^; so the complex is not in a DNA cleavage-activating state conformation. Although a recent study revealed a second conformational sate, in which the HNH domain is closer to the cleavage site^11^, the distance from the C_α_ atom of catalytic residue 840 to the attacked phosphorus is still more than 19 Å (HNH-state 2 in Supplementary Fig. 1c). Also, a certain number of nucleotides from the 20-mer non-target sequence are missing in the available crystal structures^7, 11^ (Supplementary Fig. 1). So far no atomic model has been built for the ternary complex with a full-length DNA target in the DNA cleavage-activating state. Therefore, it is worthwhile exploring new complex structures, in particular active structures in the native cleavage environment, in order to provide new insights into the molecular mechanisms of DNA cleavage by CRISPR-Cas9.

Here we use cryo-electron microscopy (cryo-EM) to determine the structure of *Streptococcus pyogenes* Cas9 (SpCas9) in the DNA cleavage-activating state. As a result, a 5.2 Å cryo-EM structure of SpCas9 in complex with sgRNA and target DNA is reported. Compared with two known HNH states of SpCas9 (Supplementary Fig. 1), this structure captures a distinct conformational state in which the HNH domain is nearest to the DNA cleavage site. EM-based molecular dynamics (MD) simulations show that residues of the nuclease domains in this structure could form cleavage-compatible conformations with the target DNA. Therefore, this study provides mechanistic insights into the DNA cleavage by Cas9, and will facilitate engineering efforts to improve the specificity and efficiency of the CRISPR-Cas9 system.

## Results

### Cryo-EM structure of the Cas9–sgRNA–DNA ternary complex

**Fig. 1:**
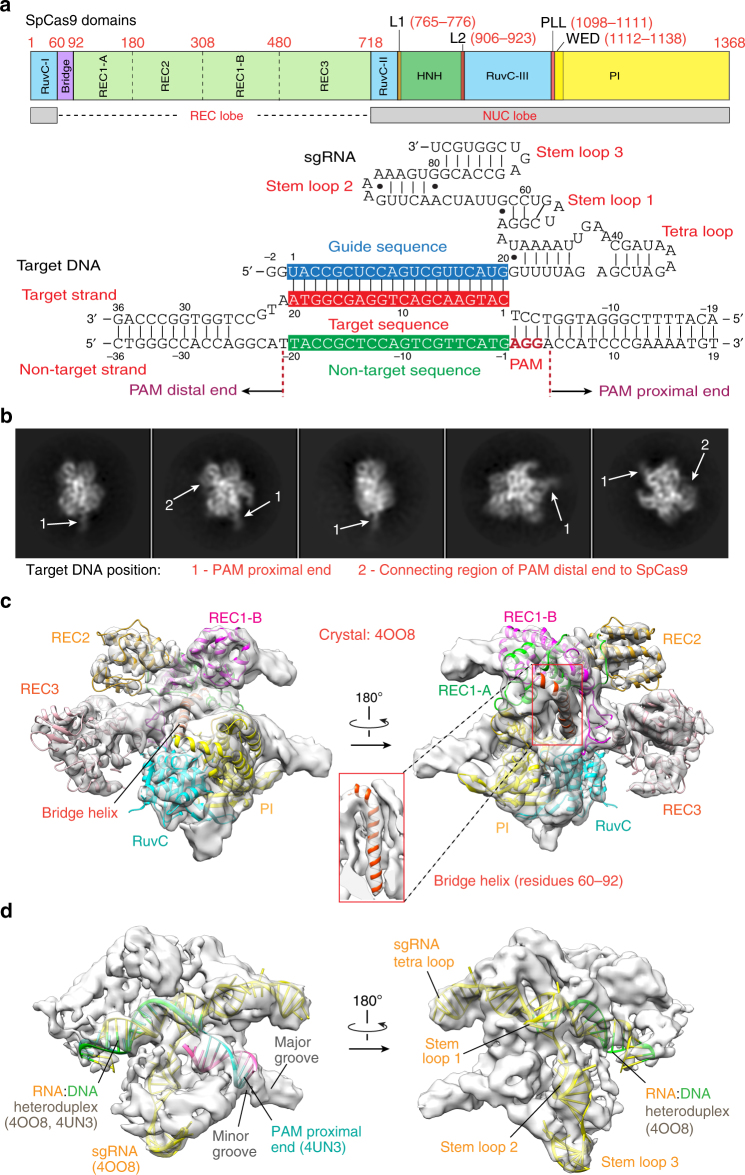
The cryo-EM structure of the ternary complex. **a** Schematic diagrams of SpCas9, 98-nt sgRNA, 55-bp target DNA. Potential Watson–Crick and non-Watson–Crick base pairs in sgRNA and DNA are indicated by lines and dots, respectively. **b** Representative 2D classes of the complex particles. The positions of the PAM proximal and PAM distal ends of the target DNA are indicated by arrows 1 and 2, respectively. **c** Rigid-body fitting of the crystal structure 4OO8 in the absence of the HNH domain, with a correlation coefficient of ~0.80. The density map at a higher contour level (~4.0*σ*) shows clearly most of the SpCas9 α-helices in the crystal structure (cartoon model). The central, small panel shows the EM density that resolves the long bridge helix. **d** The density map clearly resolves sgRNA, the RNA:DNA heteroduplex, PAM, and the PAM proximal end in the crystal structures (4OO8 and 4UN3, cartoon model). The correlation coefficient of the rigid-body fitting of 4UN3 is ~0.80

To determine the cryo-EM structure of the Cas9–sgRNA–DNA ternary complex in Fig. 1a, SpCas9 with two nuclease activity-dead mutations (D10A, H840A) was incubated with a 55-bp target DNA from the fumarylacetoacetate hydrolase (*Fah*) gene^12^ and the corresponding 98-nucleotide (nt) sgRNA to form the ternary complex (Supplementary Fig. 2). The complexes were then rapidly frozen to acquire cryo-EM images for single-particle reconstruction^13, 14^ (Supplementary Fig. 3a; Supplementary Table 1).

As illustrated by representative images in Fig. 1b, 2D class averaging shows the formation of the ternary complex. The 16-bp PAM proximal end of the DNA chain was clearly identified (arrow 1 in Fig. 1b) and weaker density likely corresponding to the connecting region of the PAM distal end to SpCas9 is also visible (arrow 2). After 3D classification and refinement (Supplementary Fig. 3b), we obtained an EM density map at an overall resolution of 5.2 Å with the Fourier shell correlation (FSC) at 0.143 (Supplementary Fig. 3c). Consistent with the 2D averaging images, the density map suggests that the PAM proximal end is in a stable, base-paired form. In contrast, no significant density was observed for the 16-bp PAM distal end, but only two small, separate density projections on the surface. Their positions are similar to that of the 10-bp PAM distal end in the recent cryo-EM structure with a 40-bp target DNA (6.0 Å resolution)^11^ (Supplementary Fig. 4), and may be the connecting regions of two unwound DNA strands to SpCas9. These observations suggest that most of the ternary complexes in the cryo-EM experiments possess a PAM distal end in a loosely flexible form, which might be caused in the process of the RNA:DNA heteroduplex formation.

**Fig. 2:**
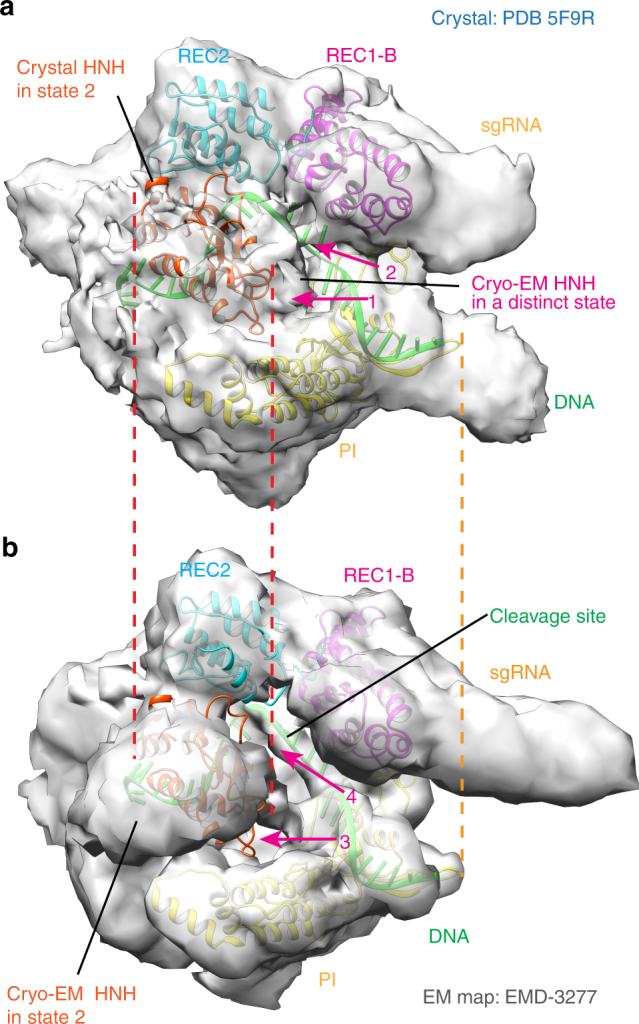
The conformational state 3 of the HNH domain revealed by the cryo-EM density map. **a** Density regions that indicate a closer contact of the HNH domain with the REC1 and PI domains (contoured at ~1.0*σ*), as indicated by arrows 1 and 2. The dashed lines indicate the boundaries of the HNH and PI domains in the fitted crystal structure (PDB 5F9R). **b** No corresponding regions in the EM density map of the ternary complex with a 40-bp target DNA (contoured at ~1.7*σ*), as indicated by arrows 3 and 4

Our ternary complex has a well-defined bilobed topology, which is fully consistent with the overall domain architecture observed in the previous crystal structures (PDB IDs 4OO8 and 4UN3)^6, 7^. Simple rigid-body fitting showed that the complex domains in the crystal structures fitted well into the EM density map. As shown in Fig. 1c, d, the density map at a higher contour level accurately resolves most of the secondary structural elements in these structures, including helices of SpCas9, the RNA:DNA heteroduplex, PAM, and the base-paired PAM proximal end. For example, most of the helices in the highest resolution (2.5 Å) crystal structure (4OO8) are located in the EM density regions with higher contour levels, and were exactly fitted into those regions (Fig. 1c), except for those in HNH, L1, and L2 regions, and one in the RuvC domain close to HNH (Supplementary Fig. 5). Meanwhile, sgRNA, the RNA:DNA heteroduplex, PAM, and the PAM proximal end in 4UN3 are also clearly resolved (Fig. 1d). For example, the major and minor grooves in the base-paired PAM proximal end were accurately identified. The clear definition of the secondary structural elements further validates the reliability of our cryo-EM 3D construction, as illustrated in Supplementary Fig. 3b. When considering that the crystal structures 4OO8 and 4UN3 are ternary complexes without the full-length non-target DNA strand, our EM map implies that the resolved helices in Fig. 1c possess similar structures in the absence and presence of the full-length non-target strand. On the other hand, when bound to the full-length non-target strand, the HNH, L1, and L2 regions and part of the RuvC domain might have different conformations from those observed in the crystal structures (Supplementary Fig. 5).

Significantly, similar to the recent study^11^, our EM map also reveals a large conformational change of the HNH domain (residues 775–906) with respect to that in 4OO8 or 4UN3 (Supplementary Fig. 1). However, the HNH domain in our map is closer to the cleavage site by contacting the PI and REC1 domains (Fig. 2a). In contrast, no comparable density is present in the cryo-EM structure at 6.0 Å resolution^11^ (Fig. 2b). We conclude that our cryo-EM experiment visualized a distinct conformational state of SpCas9 in a nearly native environment primed for the DNA cleavage (hereafter designated as HNH-state 3). So this structure provides further experimental evidence for the activation mechanism of the DNA cleavage: the ternary complex formation induces an HHN conformational change that moves its active site toward the cleavage site, and meanwhile mediates the binding of the non-target DNA strand to the RuvC active site^11, 15^. The HNH mobility appears to be an intrinsic feature of the CRISPR-Cas9 system that is required for its cleavage function.

### EM-based atomic model

As shown in Fig. 1c, d, the unambiguous placement of the non-HNH elements of the crystal structures into the density map allowed us to build atomic models for the ternary complex in the HNH-state 3 by combining homology modeling^16^, molecular dynamics flexible fitting (MDFF)^17^, and structural averaging refinement^18^. Since we did not observe significant density for the PAM distal end, we determine an atomic model for the SpCas9–sgRNA–DNA ternary complex, which includes only two base pairs of the PAM distal end linking to target/non-target sequence. Thus, the EM density-based atomic model consists of the experimental SpCas9 mutant (D10A, H840A), sgRNA, and 41 base pairs of the target DNA (Supplementary Fig. 6).

**Fig. 3:**
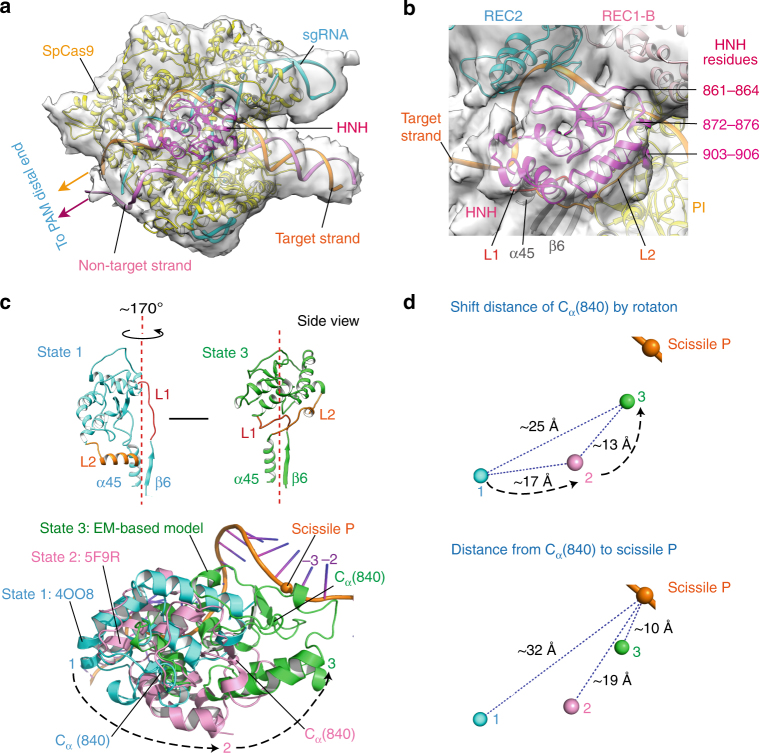
The EM-based atomic model of the ternary complex. **a** The EM density-based atomic model with the 41-bp DNA target (in cartoon). The possible positions of two DNA strands of the PAM distal end are indicated by two arrows. **b** The close contacts of the HNH domain with the REC1 and PI domains. **c** Three rotational states of the HNH domain. **d** Different state distances from the C_α_ atom of the catalytic residue 840 to the phosphorus of the scissile bond

As shown in the atomic model in Fig. 3a, the HNH domain fits well into the density regions corresponding to the HNH-state 3, and contacts the REC1 and PI domains mainly by the segments of residues 861–864, 872–876, and 903–906 (Fig. 3b). Compared to the HNH-state 1, the HNH domain as a whole rotates about 170° around an axis, roughly, which is located vertically at the middle of a β-sheet segment (β6, residues 757–764) and an α-helix (α45, residues 925–940) (Fig. 3c). To facilitate the movement, the linker L2 (residues 906–923) undergoes a helix-to-loop conformational change, similar to that of the *Staphylococcus aureus* Cas9 (SaCas9)^10^. It seems that the HNH-state 2 is an intermediate state between the HNH states 1 and 3 (Fig. 3c). Through the rotation, with respect to the HNH states 1 and 2, the HNH active site is shifted toward the cleavage site by about 25 and 13 Å, respectively (Fig. 3d), so that the distance between the C_α_ atom of residue 840 and the scissile P reduces from ~32 (state 1) and ~19 Å (state 2) to ~10 Å (state 3), respectively. These distance reductions provide evidence that our cryo-EM study did indeed capture a distinct SpCas9 state in which the HNH active site is the closest to the scissile bond of the target DNA.

**Fig. 4:**
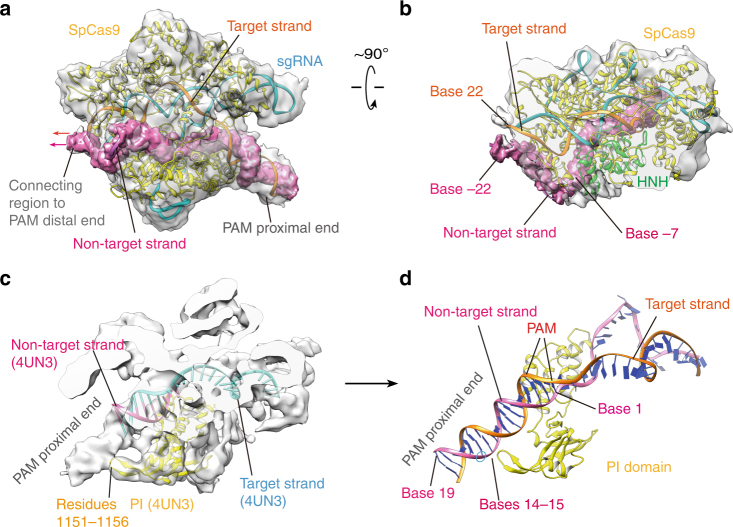
The SpCas9-bound topology of the non-target DNA strand (bases −22 to 19). **a** The EM density (contoured at ~1.0*σ*) corresponds to the non-target strand from the base-paired PAM proximal end to the connecting region of the PAM distal end to SpCas9 (in hot pink). The possible positions of the PAM distal end are indicated by arrows. For clarity, the HNH domain is not presented in the atomic model (in cartoon). **b** Top view of the density and possible connecting bases of the PAM distal end to SpCas9. **c** The EM map (contoured at ~4.0*σ*) reveals a binding interaction of the PAM proximal end with SpCas9 segment 1151–1156. The fitted atomic model is the crystal structure 4UN3 (Fig. 1c, d). **d** The bases of the non-target strand that interact with the segment 1151–1156

Our model also reveals the overall topology of the non-target strand bound to SpCas9. As shown in Figs. 1 and 3, the clear assignment of the EM density to SpCas9, sgRNA, the RNA:DNA heteroduplex, PAM, and the PAM proximal end allowed us to assign the remaining density to the first 41 bases of the 55-mer non-target strand in the model (Fig. 4a). As seen, the remaining density extends from the based-paired, PAM proximal end to the connecting region of the PAM distal end to SpCas9 in Supplementary Fig. 4. This connecting region is likely located at base −22 of the non-target strand. Because of the close contact of the HNH domain with the PI domain, about 7 bases of the 20-mer non-target sequence are enclosed in the channel formed by the HNH, RuvC and PI domains (from bases −7 to −1), including the scissile bond of the non-target strand (Fig. 4b). Therefore, about 13 bases of the non-target sequence are bound to the surface of Spcas9.

In addition, our cryo-EM structure reveals an interaction site of the 16-bp PAM proximal end with SpCas9 (Fig. 4c). Compared with our structure, the PAM proximal ends in the previously determined structures^7, 11^ are relatively short and this SpCas9–DNA interaction was not observed in those structures. By rigid-body fitting of the crystal structure (4UN3), we found that the SpCas9 protein binds to the PAM proximal end by its segment 1151–1156 in the PI domain (Fig. 4c). Since four residues of this segment are positively charged lysines, they might bind to the negatively charged, DNA backbone by electrostatic forces. The EM-based atomic model indicates that this segment interacts with bases 14–15 of the non-target strand, about 11 bases downstream PAM (Fig. 4d). Therefore, we speculate that this SpCas9–DNA interaction may play a role in the process of PAM recognition.

### MD simulations

Since the cryo-EM density map shows a new domain architecture and backbone conformations of the complex, we wanted to know whether the wild-type residues of the HNH and RuvC domains in this architecture might be able to form the cleavage-compatible conformations with the DNA strands and the Mg^2+^ ions. Because it is difficult to use the catalytic active wild-type SpCas9 protein to form a stable complex with the target DNA, structural data are not yet available for directly building atomic models of the wild-type active sites of SpCas9. Therefore, we used our EM-based atomic model as an template to build the wild-type active site models. We changed its two activity-dead mutations into the wild-type residues (Asp10, His840) and then refined the residue positions by large-scale MD simulations^19^, in order to predict the most probable conformations of the wild-type HNH and RuvC residues with the target DNA and Mg^2+^ ions (Supplementary Fig. 6).

**Fig. 5:**
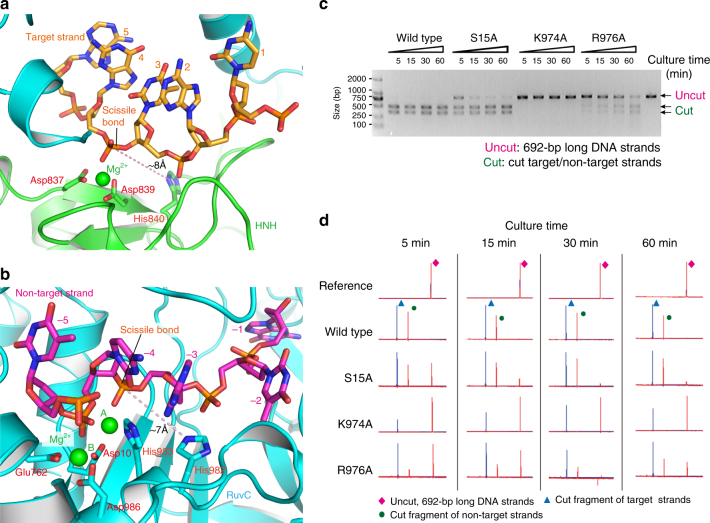
The MD models of the wild-type HNH and RuvC active sites. **a** Close-up view of the HNH active site in complex with the target strand and one Mg^2+^ ion. **b** Close-up view of the RuvC active site in complex with the non-target strand and two Mg^2+^ ions. **c** DNA cleavage activities of the wild-type SpCas9 and three mutants detected by agarose gel electrophoresis. **d** DNA cleavage activities detected by capillary electrophoresis. The reference is the uncut, 692-bp long DNA strands. Data are shown for one representative experiment from three independent experiments with similar results

Interestingly, the MD simulations showed that two wild-type nuclease domains in the experimental cryo-EM architecture may form cleavage-compatible conformations with the target DNA for using the conserved HNH and RuvC catalytic mechanisms. Previous biochemical studies have suggested that the HNH domain uses a well-established, one-metal-ion hydrolysis mechanism to split the phosphodiester bond (P-O) of the target strand between bases 3 and 4 upstream of PAM^4, 20, 21^. Consistent with this mechanism, the MD model shows that the phosphate group between bases 3 and 4 binds to the HNH catalytic cleft enclosed by Asp837, Asp839, and His840 (Fig. 5a). A Mg^2+^ ion binds to the carboxyl groups of Asp837 and Asp839 in this model and coordinates with the oxygen atoms of the phosphate group; besides, the N_δ_ atom of the general base His840 points toward the phosphate group, which could facilitate a nucleophilic attack with a water molecule to target the attacked phosphorus^22^. The distance from the N_δ_ atom to the attacked phosphorus is ~8 Å, which is long enough to could accommodate 1–2 water molecule(s) for hydrolysis. Further MD simulation confirmed that water molecules could stay stable at the HNH active site and on average, about 19 water molecules were present around the bound Mg^2+^ within 7 Å in the simulations (Supplementary Fig. 7a, b).

Meanwhile, consistent with the two-metal-ion hydrolysis mechanism^6, 23, 24^, the MD model shows that two Mg^2+^ ions are bound to the carboxyl groups of Asp10, Glu762, Asp986. (Fig. 5b). The Mg^2+^-bound phosphate group between bases −4 and −3 of the non-target strand is the nearest one to the possible general base His983 or His982 for the hydrolysis. This suggests that the cleavage may take place at the P-O3′ bond of bases −4 and −3 by the two-metal-ion mechanism^4, 20, 21^. Similar to what was observed for the HNH domain, the MD simulations showed that about 14 water molecules could stay in the RuvC active site around the center of two bound Mg^2+^ ions (within 10 Å) (Supplementary Fig. 7a, c).

### Structure-based analysis of SpCas9 mutants

To further verify the above structural model by MD, we also used this model and Rosetta program^25^ to predict RuvC residues that are important for the DNA binding. The structure-based prediction suggested that residues of the RuvC domain might make significant contributions to the binding (Supplementary Fig. 8a). Accordingly, we expressed seven SpCas9 mutants (S15A, Q920A, S964A, R967A, K974A, R976A, and N1317A) and examined their DNA cleavage activities.

We found that three RuvC mutants (S15A, K974A, R976A) were affected in their cleavage activities: S15A reduces the DNA cleavage rate, and K974A and R976A disrupt or reduce the DNA cleavage ratio (Fig. 5c), which is in agreement with a previous study^26^. Capillary electrophoresis analysis indicated that the mutations only affected the cleavage of the non-target strand, but not that of the target strand (Fig. 5d), which supports that the MD model represents the bound state of the non-target strand to the RuvC domain. Indeed, our model shows that all three residues are located in the vicinity of the RuvC active site and interact with the non-target strand by hydrogen bonding (Supplementary Fig. 8b), and thus provides a structural explanation for their crucial roles in the DNA cleavage^26^. However, to fully understand the DNA cleavage mechanisms by SpCas9, further ternary complex structures at higher resolutions are needed in the future.

## Discussion

Although the CRISPR-Cas9 technology has been widely used for precision genome engineering, structural data for its DNA cleavage step are still incomplete. In this study, we have determined a cryo-EM structure for the SpCas9–sgRNA–DNA ternary complex at 5.2 Å resolution, and visualized a conformational state of SpCas9, in which the HNH active site is the closest to the target DNA cleavage site. The atomic model and the EM-based MD simulations strongly support that this cryo-EM structure represents a DNA cleavage-activating architecture of SpCas9, and thereby marks an important step toward elucidating how the Cas9 nuclease domains form the catalytic conformations commitment for splitting the DNA strands. Thus, our study provides valuable insights into the DNA cleavage mechanism by the CRISPR-Cas9 system, and opens new opportunities for improvement of its specificity and efficiency by exploiting potential Cas9–DNA interactions.

## Methods

### SpCas9 protein expression and preparation of nucleic acids

The sequence encoding SpCas9 and activity-dead SpCas9 (dCas9) were cloned into a pET-28a expression vector between the *Nde*I and *Xho*I sites, followed a His_6_-GST tag and a TEV protease cleavage site at N-terminal. The primer sequences for constructing the plasmids are listed in Supplementary Table 2. These two proteins were expressed and purified by Novoprotein Co. Ltd. In brief, the expression vectors were transformed into *Escherichia coli* strain BL21 (DE3) (Tiangen Biotech), cultured in LB medium with 0.1 mM IPTG at 16 °C for 20 h for protein expression. The cells were harvested and lysed two times using a French press (JN-3000 PLUS, JNBIO) at 10,000 psi in lysis buffer (20 mM HEPES, pH 7.5, 500 mM KCl) at 4 °C. Clarified lysate was bound in batch to Ni-NTA agarose (Qiagen), washed with washing buffer (lysis buffer with 30 mM imidazole, pH 7.5), and eluted with elution buffer (lysis buffer with 100 mM imidazole, pH 7.5). The proteins were then incubated overnight at 4 °C with TEV protease to remove the His_6_-GST tag. After further purification by heparin affinity chromatography, Sepharose HiTrap (GE Healthcare) and HiLoad Superdex 200 16/60 columns (GE Healthcare), the purified SpCas9 and dCas9 proteins were concentrated to 10.8 mg mL^−1^ and 15.4 mg mL^−1^, respectively, in Cas9 storage buffer (20 mM HEPES, pH 7.5, 150 mM KCl and 1 mM TCEP)^4^, and stored in −80 °C.

All the other SpCas9 mutants were introduced by sited-directed PCR and were subcloned into a pET-21a vector with His_6_-tag at N-terminal. The primer sequences for constructing the SpCas9 mutants are listed in Supplementary Table 2. These mutant proteins were expressed in *E. coli* strain Rosetta (Tiangen Biotech) with 0.1 mM IPTG incubation, and were purified by chromatography with Ni-NTA agarose (Qiagen) on BioLogic LP system (Bio-Rad), similar to the purification of the wild-type SpCas9. The eluent proteins were concentrated to 1–2 mg mL^−1^ by 100,000 MWCO centrifugal filter (Merck Millipore) in the Cas9 storage buffer with 50% glycine, and stored in −20 °C.

The 98-nt sgRNA was in vitro transcribed using PCR-generated DNA templates by MEGAshortscript T7 Transcription Kit (Thermo Fisher), and was purified by MEGAclear Transcription Clean-Up Kit (Thermo Fisher). The 55-mer target/non-target DNA strands were commercially synthetized (Sangon Technology), pre-annealed to double-strand DNA substrate (dsDNA) by heating to 95 °C and then slowly cooling down to room temperature in NEB buffer 2.1. The primer sequences used for sgRNA transcription are listed in Supplementary Table 2.

### Cryo-EM

Purified dCas9 protein was mixed with the 98-nt sgRNA and 55-bp target DNA at a molar ratio of 1:1.2:1.5 to form the Cas9–sgRNA–DNA complex. The ternary complex was prepared in three steps: first, dCas9 protein was incubated with sgRNA in the reaction buffer (20 mM Tris-Cl (pH 7.5), 100 mM KCl, 5 mM MgCl_2_, 1 mM DTT)^5^ at 37 °C for 10 min. Second, dsDNA was added and incubated for another hour; third, complex solution was cultured at 18 °C overnight to disperse the proteins. The final concentrations of dCas9, sgRNA, and DNA were 2.0, 2.4, and 3.0 μM, respectively. The formation of the ternary complex was confirmed by agarose gel electrophoresis.

The cryo-EM frozen sample was prepared by a standard plunge freezing procedure^14^. In brief, 2.2 μL dispersed complex solution was loaded onto a glow-discharged holey carbon grid (Quantifoil, 1.2 μm hole size, 200 meshes) and plunge-frozen in liquid ethane using Vitrobot (FEI) system. The sample was blotted for 4 s in the environment of 16 °C and 100% humidity. Then, the grids with frozen sample were imaged on a Titan Krios (FEI) EM operated at 300 kV, and collected using a K2 Summit camera (Gatan). The micrographs were recorded at a nominal magnification of ×18,000 (calibrated pixel size of 1.3 Å on the specimen) using low-dose exposure (10 e/s pixel^2^), exposure time 7.6 s, leading to a total accumulated dose of 45 e Å^−2^. Each of the images recorded by K2 was with a random defocus ranging from −1.3 to −3.5 μm and was fractionated into 38 subframes.

### Single-particle 3D reconstruction

All recorded cryo-EM images were aligned with whole-image motion correction^14^ and the subframes were summed together to a single micrograph for subsequent calculation. The defocus value of each micrograph was estimated by CTFFIND3^27^. As illustrated in Supplementary Fig. 3b, about 300,000 particles were picked from 592 micrographs and analyzed in the single-particle 3D reconstruction using RELION (version 1.4)^28^. First, four rounds of 2D reference-free alignment and classification (40 iterations) were carried out. A subset of ~67,000 particles from good 2D classes were selected for the unsupervised 3D classification using five classes. The crystal structure (PDB ID 4UN3) in the HNH-state 1 and in complex with a 5-bp PAM proximal end was low-pass filtered to 60 Å and then used as the reference. Then, the largest class with ~86% particles was selected for the subsequent high-resolution 3D refinement. The 3D auto-refine procedure yielded a unmasked density map at an overall resolution of 7.28 Å according to the gold-standard FSC = 0.143 criterion. Finally, the RELION post-processing with auto-masking and a B-factor of −80 Å^2^ was carried out to sharpen the map to the resolution of 5.20 Å.

### Building and refinement of atomic models

The atomic model of the ternary complex was built based on the cryo-EM density map and the crystal structures of SpCas9 in complex with sgRNA and short target DNAs (PDB IDs 4OO8 and 4UN3), with a series of modeling methods described in Supplementary Fig. 6. The crystal structure in 4OO8 was used to build the initial model of the full-length SpCas9 by homology modeling with MODELLER^16^. The sgRNA and target DNA in 4UN3 were used to build the initial model of the 98-nt sgRNA and the 41-bp target DNA by base replacement and sequence extension. The non-target sequence in the RuvC active site was placed by structural alignment using the structure of a typical RuvC domain (PDB 4LD0)^29^ as the template. The initial position of the HNH domain was placed according to a reference structure (PDB 2QNC)^30^ and the cryo-EM density. The initial all-atom model of the ternary complex was constructed by assembling the full-length SpCas9 with the experimental sgRNA, target DNA according to their relative positions in 4OO8 and 4UN3. The Mg^2+^ ions in the HNH and RuvC active sites were placed using their corresponding positions in 2QNC and 4LD0 as the templates, respectively. This complex model was then docked into the cryo-EM density map using the COLORES program^31^, and subsequently flexibly fitted into the map using the typical MDFF protocols^17^ with the program NAMD (Version 2.10)^32^. To prevent overfitting, harmonic restraints were applied to relevant internal coordinates to preserve the secondary structures of SpCas9 and the sgRNA:DNA heteroduplex. To improve the final model, several rounds of structural refinements were carried out by Rosetta macromolecular modeling suite^33^.

The MD model was built with the EM-based atomic model by changing its two activity-dead mutations (Ala10, Ala840) into the wild-type residues (Asp10, His840). Then, this complex model was refined by the MD-based structure averaging method^19^. To the end, MD simulations with explicit water model were carried out (see the following subsection for simulation details). In the simulations, the atomic coordinates of the ternary complex were saved at 20-ps intervals. The final atomic model of the wild-type ternary complex was obtained by averaging the MD snapshot structures from four independent simulations of ~80 ns. The structures of the complex and its domains were visualized via UCSF chimera^34^ and PyMOL (http://pymol.org).

### MD simulation with explicit water model

The MD simulations with explicit water model were conducted using NAMD^32^. The CHARMM36 force field^35^ and the TIP3P water^36^ model were employed to model the simulation system of the ternary complex with the water solvent. To build a simulation system, the all-atom structure of the ternary complex was solvated in the center of a cubic box of water with a minimum distance of 12 Å from the complex surface to the edge of the box. The Na^+^ and Cl^−^ ions were used to mimic an ionic concentration of 0.15 M in the system, including certain number of additional Na^+^ ions that neutralizes the net negative charge of the complex. Periodic boundary conditions were used in the simulations, i.e., the Van der Waals interactions were treated with a cut-off distance of 10 Å using a smooth switching function from 8 Å, the electrostatic interactions were calculated with particle mesh Ewald (PME) method using a local interaction distance of 10 Å, the SHAKE algorithm was employed to constrain bonds involving hydrogen atom, and thereby a time step of 2.0 fs was used. The simulations were performed in the isobaric-isothermal (NPT) ensemble, at a constant pressure of 1 bar and a constant temperature of 298 K controlled by Langevin dynamics.

### Computational analysis of SpCas9–DNA interface

The RosettaDNA program in the Rosetta macromolecular modeling suite^33^ was used to identify the key residues of the RuvC domain that interact with the 20-mer non-target sequence, based on the MD-refined model of the wild-type ternary complex. For each residue at the interface of the RuvC domain and the non-target sequence, the extent of optimal for affinity was calculated with RosettaDNA^22^. As illustrated in Supplementary Fig. 8a, those interface residues with higher extents were considered to be significant for the RuvC binding to the non-target sequence, and thereby important for the cleavage activity of the RuvC nuclease domain.

### DNA cleavage assay

The substrate DNA for the DNA cleavage assay was amplified by PCR with a pair of fluorescent labeled primers, thus, the target DNA strand was labeled by Fam and the non-target strand was labeled by Rox. To test the cleavage activity of SpCas9 mutants, the substrate DNA fragments (200 ng or ~0.6 pmol) was incubated with equal molar SpCas9/SpCas9 mutant-sgRNA duplex for 5, 15, 30, or 60 min at 37 °C in SpCas9 cleavage buffer (20 mM Tris-Cl, pH 7.5, 100 mM KCl, 5 mM MgCl_2_, 1 mM DTT, 5% glycerol)^5^. The reactions were terminated by heating the solution at 70 °C for 10 min. Half of the cleavage products were resolved by electrophoresis on 1% agarose gel stained with DNA_GREEN_ (TIANDZ) and 1/10 of the products were mixed with highly deionized-formamide (Hidi) and analyzed by capillary electrophoresis.

### Data availability

The cryo-EM map of the SpCas9–sgRNA–DNA ternary complex is deposited in the Electron Microscopy Data Bank under accession code EMD-8236. The atomic coordinates of the complex are deposited in the Protein Data Bank under the accession code 5Y36. Other data are available from the corresponding authors upon reasonable request.

## Electronic supplementary material

Supplementary Information
