# Supplementary material for: Structural insights into DNA cleavage activation of CRISPR-Cas9 system
Source: Nat Commun. 2017 Nov 9;8:1375. doi: 10.1038/s41467-017-01496-2 (PMC5680257; doi:10.1038/s41467-017-01496-2)
Supplement: Supplementary file 1 — Supplementary Information [file 41467_2017_1496_MOESM1_ESM.pdf]

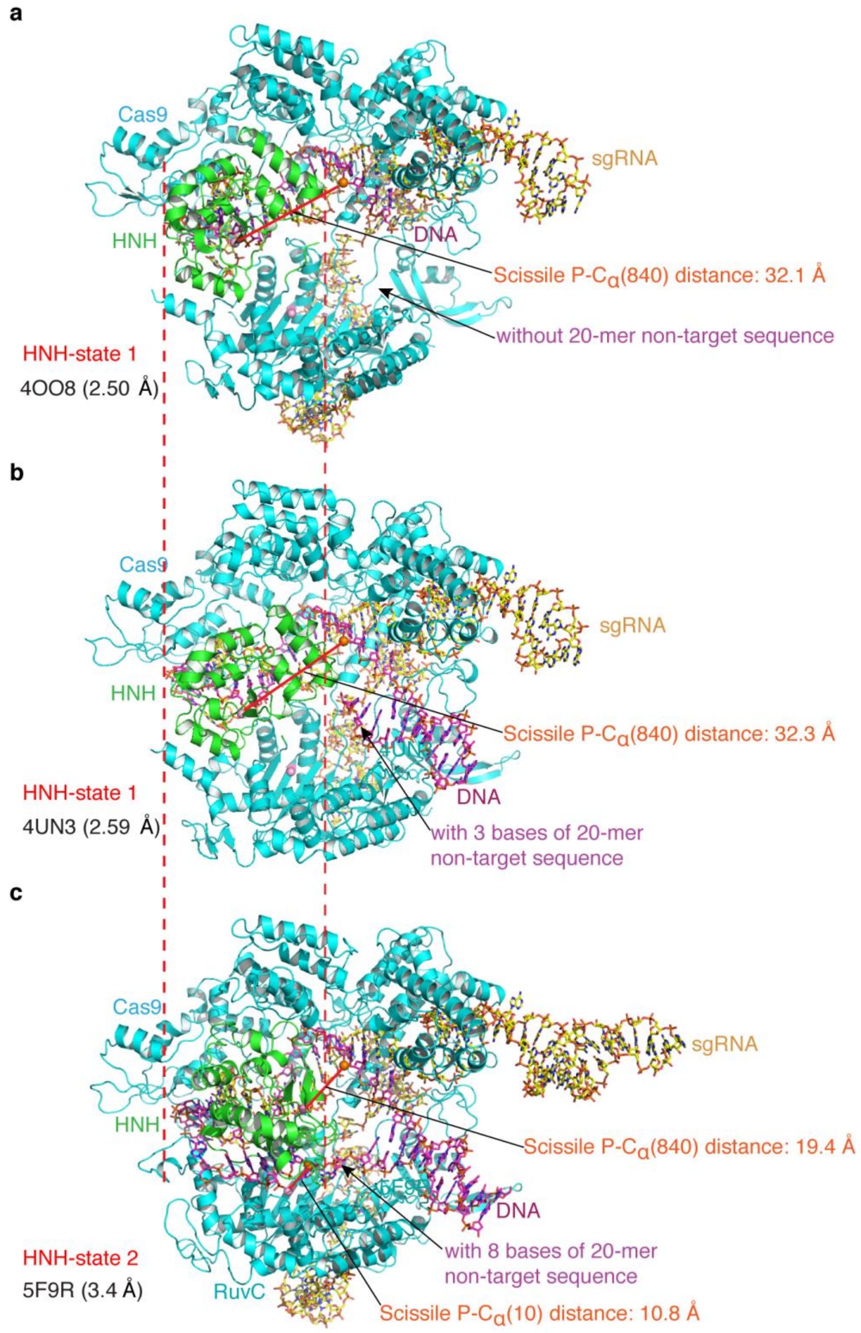


**Supplementary Figure 1 | Two distinct conformational states of the HNH domain in crystal structures. a** HNH-state 1 in PDB 4OO8, in which the distance from the $C_\alpha$ atom of the HNH catalytic residue 840 to the attacked phosphorus (scissile P) of the target strand is 32.1 Å. The dashed lines indicate the boundary of the HNH domain. **b** HNH-state 1 in PDB 4UN3, in which the distance from HNH-$C_\alpha$(840) to its scissile P is 32.3 Å. **c** HNH-state 2 in PDB 5F9R, in which the distance from HNH-$C_\alpha$(840) to its scissile P is 19.4 Å, and that from RuvC-$C_\alpha$(10) to its scissile P of the non-target strand is 10.8 Å.

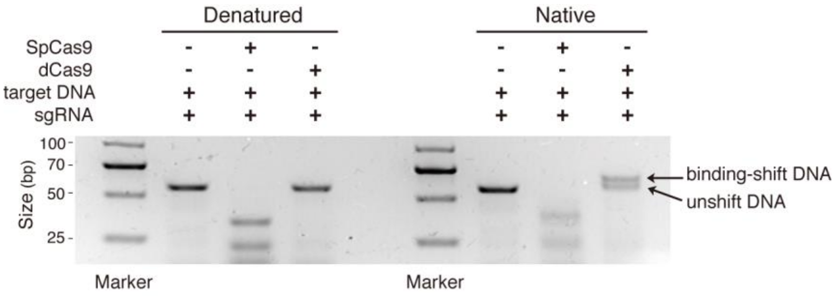


**Supplementary Figure 2 | Detection of the formation of the SpCas9-sgRNA-DNA ternary complex by agarose gel electrophoresis.** To ensure the ternary complex formation, a concentration ratio of 1:1.2:1.5 was used for SpCas9/dCas9(D10A, H840A), sgRNA and target DNA in the mixture. The electrophoresis was carried out after the mixture was cultured and dispersed overnight. Denatured: the mixture solution was heated at 70 °C for 10 min before electrophoresis to inactivate and disassociate the complexes. Native: electrophoresis without denaturation. In the last lane, the 'binding-shift' band indicates the target DNAs bound to SpCas9-sgRNA; and the 'unshift' band corresponds the remaining DNAs. Data are shown for one representative experiment from three independent experiments with similar results.

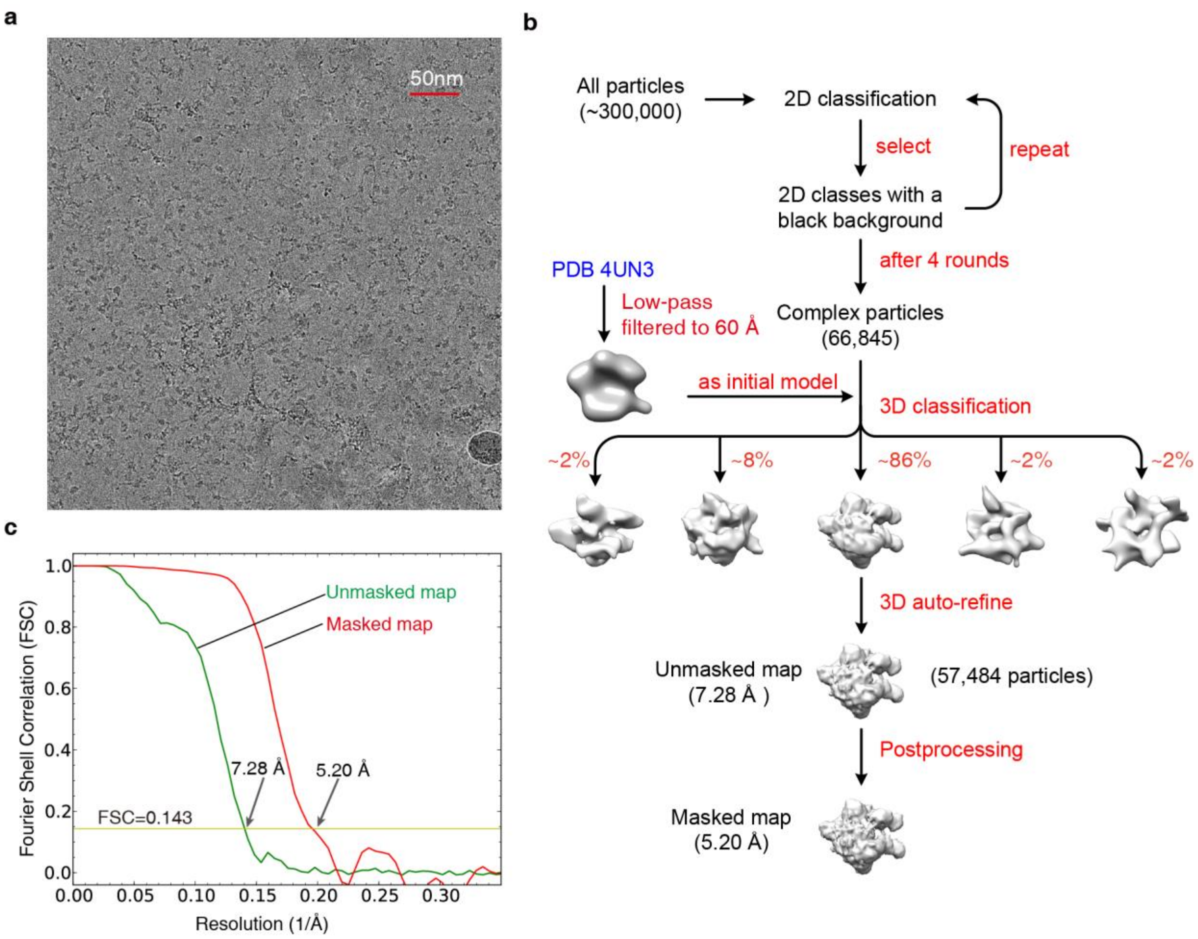


**Supplementary Figure 3 | Single-particle cryo-EM study of the SpCas9-sgRNA-DNA ternary complex. a** A typical raw micrograph of the ternary complex in the vitreous ice (scale bar, 50 nm). All cryo-EM images were collected on a Titan microscope using a K2 Summit direct detection device camera. **b** Flowchart for the single-particle 3D reconstruction with RELION. **c** FSC curves for the EM density maps between two independently refined halves, before and after post-processing in RELION.

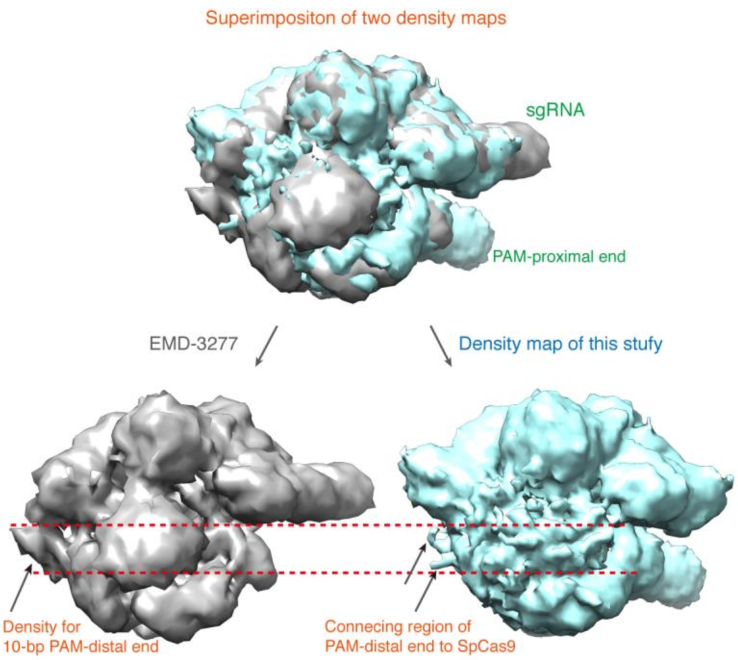


**Supplementary Figure 4 | Superimposition of cryo-EM density maps of the ternary complex.** The superimposition of our EM density map on (contoured at ~1.0σ) a recent EM structure with a 40-bp target DNA (EMD-3277, contoured at ~1.7σ) reveals relatively small density regions that may correspond to the connecting region of the PAM-distal end to SpCas9.

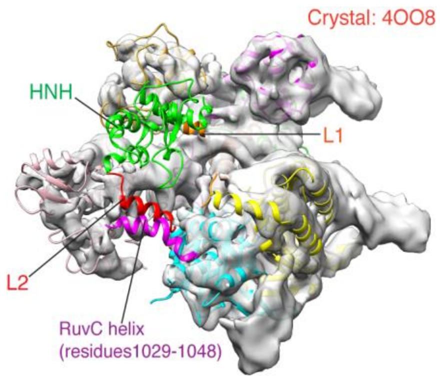


**Supplementary Figure 5 | Rigid-body fitting of the crystal structure 4OO8 with the HNH domain.** The correlation coefficient of the rigid-body fitting is ~0.80. The density map is shown at the contour level of ~4.0σ. Except for the helices in HNH, L1 and L2, and one in RuvC (residues 1029-1048), other helices in 4OO8 could be well resolved, as shown in Fig. 1c.

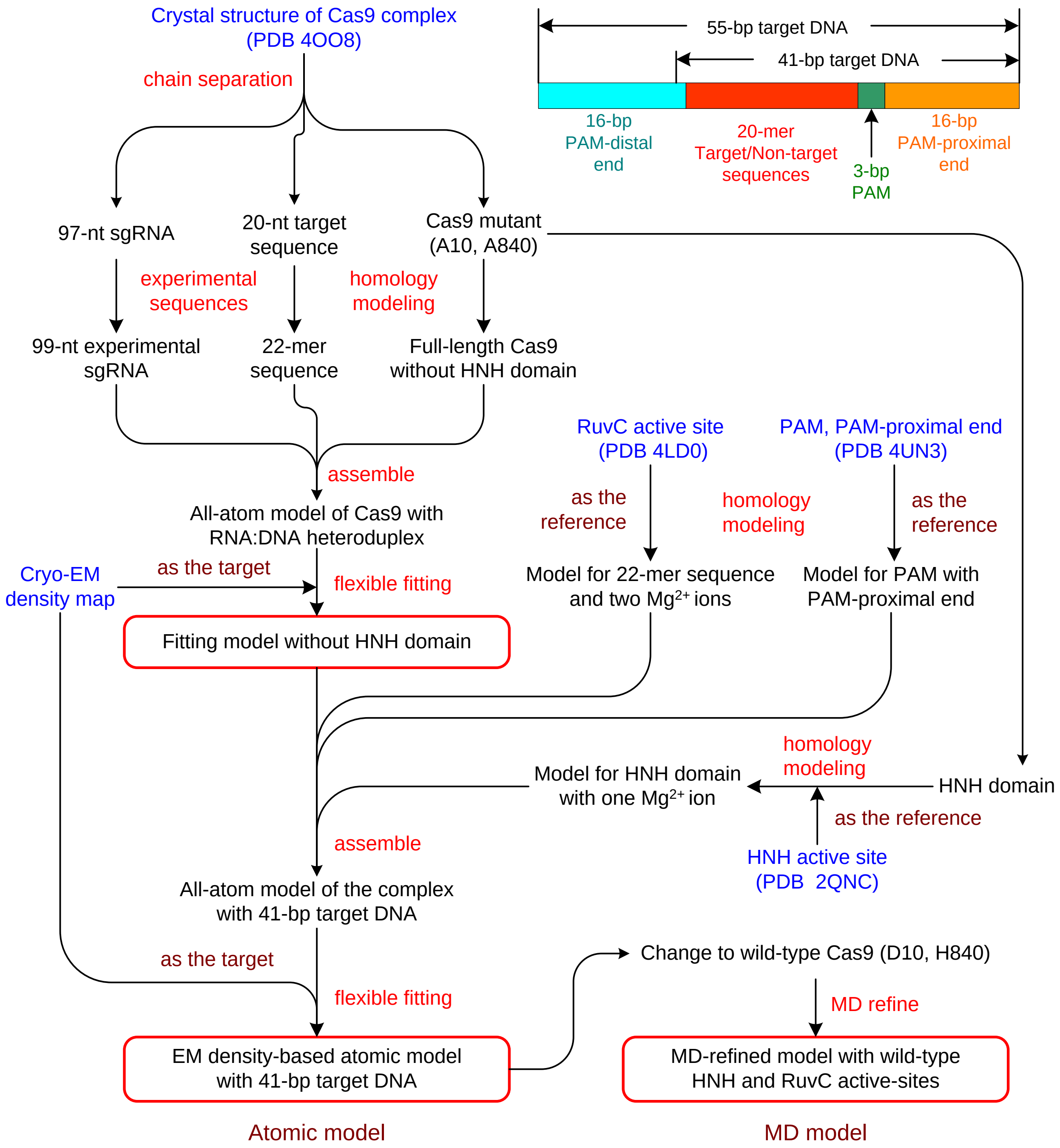


**Supplementary Figure 6 | Flowchart for the building and refinement of atomic models.** The atomic model for the ternary complex possesses the experimental SpCas9 mutant (D10A, H840A) and 41 base pairs of the 55-bp target DNA (41-bp target DNA). The MD model for the ternary complex with the wild-type SpCas9 was constructed with the EM-based atomic model, and then refined by large-scale MD simulations with explicit water solvent.

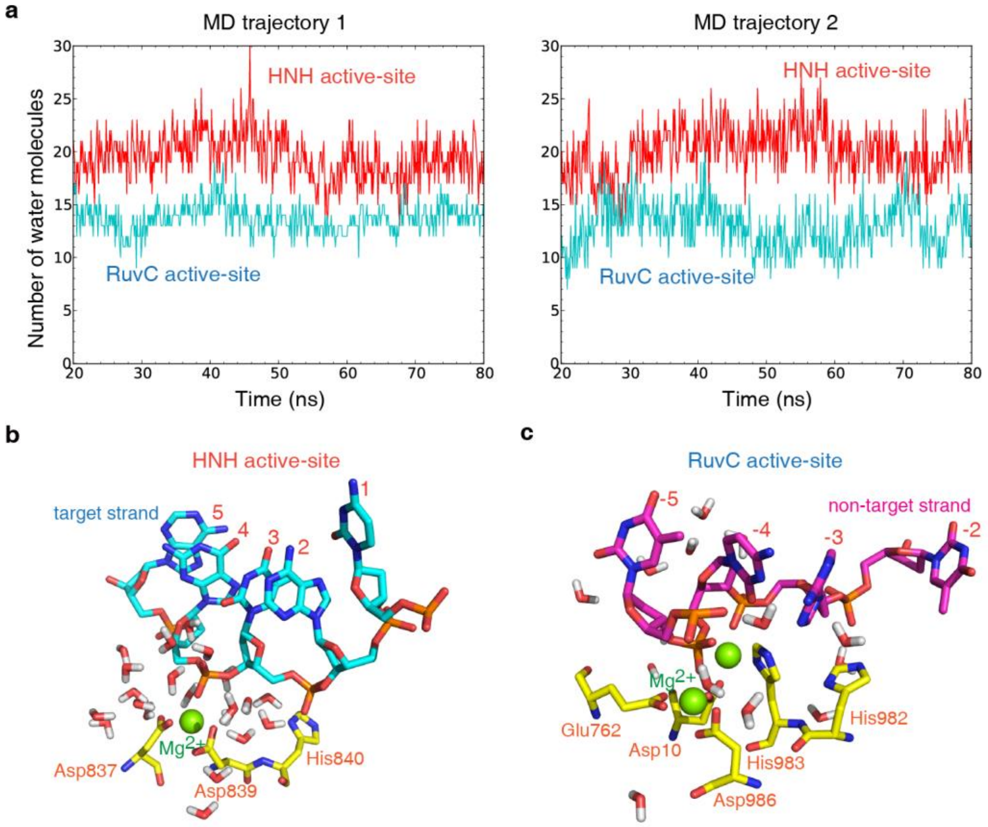


**Supplementary Figure 7 | MD simulations of the ternary complex with explicit solvent**. **a** Time-dependent number of water molecules in the wild-type HNH and RuvC active-sites in two independent MD simulations of 80 nanoseconds (ns). **b, c** Snapshots of water molecules in the HNH active-site (21 molecules within 7 Å around $Mg^{2+}$) and the RuvC active-site (14 molecules within 10 Å around two $Mg^{2+}$ ions) at 50 ns from MD trajectory 2.

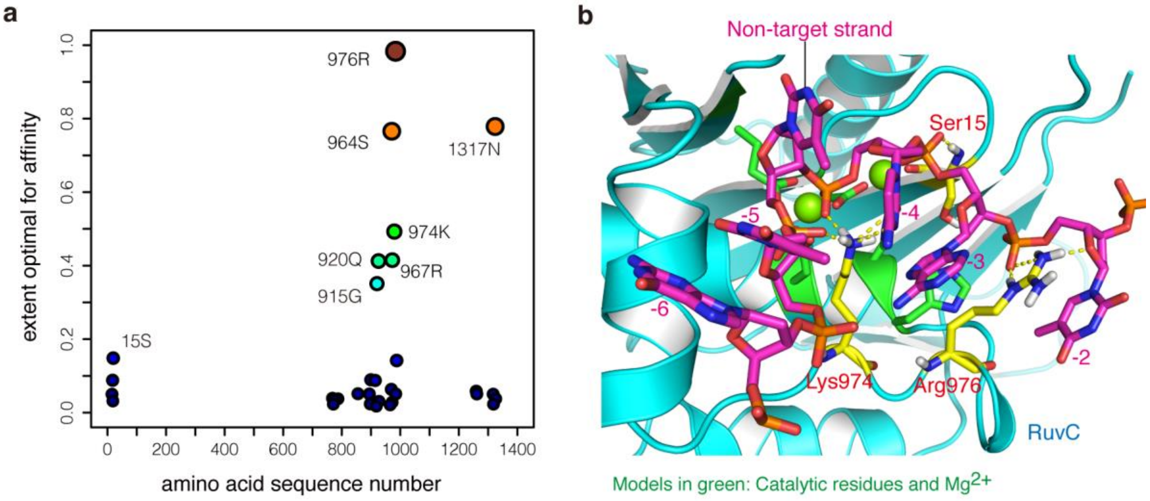


**Supplementary Figure 8 | Key RuvC residues for the binding to the non-target strand. a** Structure-based prediction of key residues using the MD-refined atomic model and the Rosetta program. The radius of each circle is proportional to the computational affinity score of the corresponding residue to the non-target DNA strand. **b** Hydrogen bonding of three key RuvC residues (in yellow) with the non-target strand in the MD model.

a

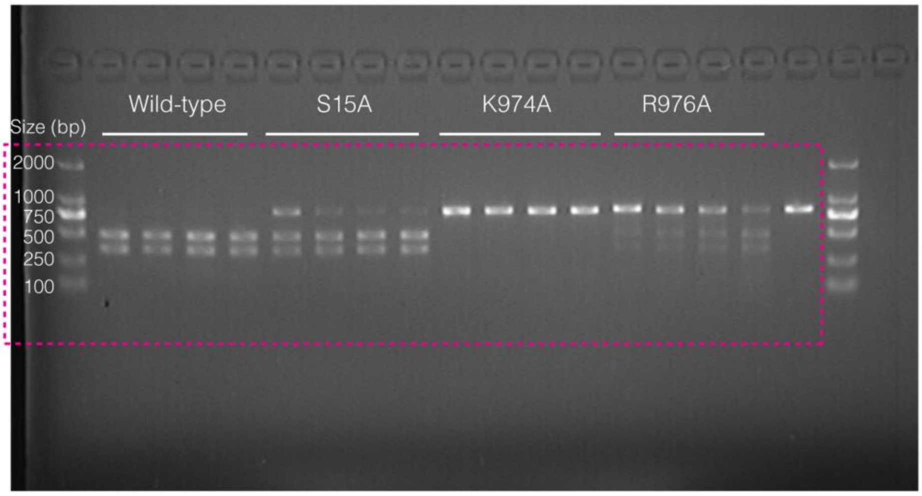


b

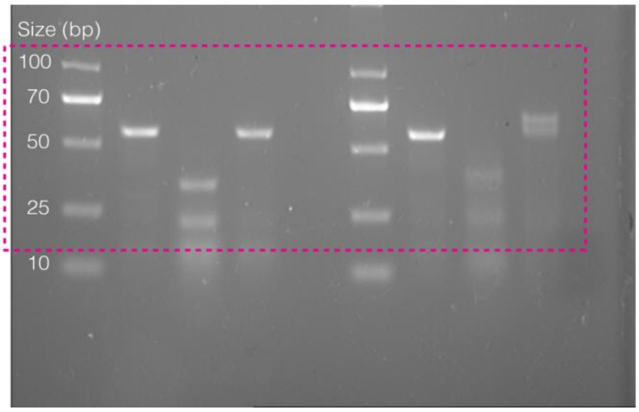


**Supplementary Figure 9 | The uncropped images of agarose gel electrophoresis. a** The uncropped image corresponding to Fig. 5c. The magenta box shows the region presented in the figure. **b** The uncropped image corresponding to Supplementary Fig. 2. The magenta box shows the region presented in the figure.

**Supplementary Table 1 | Parameters of cryo-EM data collection and 3D reconstruction**

| | |
|---|---|
| **Data collection** | |
| Micrographs | 592 |
| Voltage (kV) | 300 |
| Pixel size (Å) | 1.3 |
| Total electron dose ($e^{-}$ $Å^{-2}$) | 45 |
| Exposure time (s) | 7.6 |
| Nominal magnification | 18000× |
| Defocus range (μm) | -1.3 to -3.5 |
| **3D reconstruction** | |
| Final particles | 57484 |
| Resolution (Å) | 5.20 |
| Accuracy of rotation (degree) | 5.12 |
| Accuracy of translation (pixel) | 1.83 |
| B-factor ($Å^2$) | -80.0 |

**Supplementary Table 2 | Primer sequences used in this study**

| Primer names | Sequences (5'→3') |
|---|---|
| *Amplification of SpCas9 coding sequence* | |
| Cas9-Half1F | CGCGCGGCAGCCATATGGAAAATCTCTACTTCCAAGGCG |
| Cas9-Half1R | CAGTTTTCTTGACAGCCGCC |
| Cas9-Half2F | GGCGGCTGTCAAGAAAACTG |
| Cas9-Half2R | TGGTGGTGGTGCTCGAGTCAGTCTCCACCGAGC |
| *Construction of the dCas9 mutant(D10A, H840A)* | |
| dCas-upF | GGAATTGTGAGCGGATAACAATTCC |
| dCas-10F | ATTGGGCTCGCTATCGGCACAAACAGCG |
| dCas-10R | CGCTGTTTGTGCCGATAGCGAGCCCAAT |
| dCas-840F | GACTACGACGTGGATGCTATCGTGCCCCAGT |
| dCas-840R | ACTGGGGCACGATAGCATCCACGTCGTAGTC |
| *Construction of other SpCas9 mutants* | |
| cas9 mut upF | GCGAAATTAATACGACTCACTATAGG |
| cas9 mut upR | CCGGGATTTGGACAGCC |
| 15R | GCCGACGGCGTTTGTGCCGATATCG |
| 15F | ACAAACGCCGTCGGCTGGGCCGT |
| cas9 mut downF | GGCGGCTGTCAAGAAAACTG |
| cas9 mut downR | TTAGCGTCGGCGAGGATC |
| 974F | TTTATGCGGTGAGAGAGATCAACAATTACC |
| 974R | CTCTCTCACCGCATAAAACTGAAAGTCCT |
| 976F | TAAGGTGGCAGAGATCAACAATTACCACC |
| 976R | GATCTCTGCCACCTTATAAAACTGAAAGTCC |
| *sgRNA in vitro transcription* | |
| gRNA-F | TAATACGACTCACTATAGGTACCGCTCCAGTCGTTCATGGTTTTAGAGCT AGAAATAGCAAGTTAAAAT |
| gRNA-R | AGCACCGACTCGGTGCCACT |
| *DNA cleavage* | |
| Invitro Cut F (Fam) | CTGTGAAGTCAACTTGTCAATCC |
| Invitro Cut R (Rox) | CCCTCCATGTACAGCTCTTCAT |
